# Combinatorial nanodot stripe assay to systematically study cell haptotaxis

**DOI:** 10.1038/s41378-020-00223-0

**Published:** 2020-12-14

**Authors:** Mcolisi Dlamini, Timothy E. Kennedy, David Juncker

**Affiliations:** 1 Biomedical Engineering Department, McGill University, 3775 University Street, Montréal, QC H3A 2B4 Canada; 2 McGill Genome Centre, 740 Dr. Penfield Avenue, Montréal, QC H3A 0G1 Canada; 3 McGill Program in Neuroengineering, Montréal, QC Canada; 4 Department of Neurology and Neurosurgery, McGill University, 3801 University Street, Montréal, QC H3A 2B4 Canada

**Keywords:** Nanofabrication and nanopatterning, Nanoscale materials

## Abstract

Haptotaxis is critical to cell guidance and development and has been studied in vitro using either gradients or stripe assays that present a binary choice between full and zero coverage of a protein cue. However, stripes offer only a choice between extremes, while for gradients, cell receptor saturation, migration history, and directional persistence confound the interpretation of cellular responses. Here, we introduce nanodot stripe assays (NSAs) formed by adjacent stripes of nanodot arrays with different surface coverage. Twenty-one pairwise combinations were designed using 0, 1, 3, 10, 30, 44 and 100% stripes and were patterned with 200 × 200, 400 × 400 or 800 × 800 nm^2^ nanodots. We studied the migration choices of C2C12 myoblasts that express neogenin on NSAs (and three-step gradients) of netrin-1. The reference surface between the nanodots was backfilled with a mixture of polyethylene glycol and poly-d-lysine to minimize nonspecific cell response. Unexpectedly, cell response was independent of nanodot size. Relative to a 0% stripe, cells increasingly chose the high-density stripe with up to ~90% of cells on stripes with 10% coverage and higher. Cell preference for higher vs. lower netrin-1 coverage was observed only for coverage ratios >2.3, with cell preference plateauing at ~80% for ratios ≥4. The combinatorial NSA enables quantitative studies of cell haptotaxis over the full range of surface coverages and ratios and provides a means to elucidate haptotactic mechanisms.

## Cytology: Nanodot stripe assay for studying cell haptotaxis

Critical to cell guidance and development, haptotaxis is directional cell movement in response to an adhesive substrate, and a method has been developed using nanodot stripe assays (NSAs) to systematically study haptotaxis that supersedes conventional stripe assays. Hitherto, haptotaxis has been studied in vitro using standard stripe assays, which offer only a binary choice between full or zero response to a protein: the assays measure cellular responses under extreme conditions that do not reflect in vivo situations. However, a group headed by David Juncker at McGill University, Canada has succeeded in producing NSAs, which are formed by adjacent stripes of nanodot arrays, that in addition to the extremes, include 5 intermediate surface densities. The team used a combination of 21 NSAs of the Netrin-1 protein to study the migration choices of cells to both the extreme and 20 intermediate conditions. The authors believe their approach can better elucidate haptotactic choices and mechanisms.

## Introduction

Cell migration is essential for many biological processes including angiogenesis^1^, immune responses^2^, tissue repair^3^, cancer metastasis^4^, and embryonic development^5^. Cells migrate directionally in response to signals transduced from soluble and surface-bound cues that are present in their microenvironment via chemotaxis^6^ and haptotaxis^7,8^, respectively. For instance, fibroblasts navigate on surface-bound gradients of extracellular molecules at the interface of healthy and fibrotic tissues^3^. During embryogenesis, a gradient of the protein netrin-1 has been proposed to direct the haptotactic extension of commissural axons towards the floor plate of the developing spinal cord^9–11^. Well-characterized haptotactic cell navigation commences with cellular transmembrane receptors binding to extracellular protein ligands via cell adhesion molecules such as integrins. These ligand−receptor junctions establish macromolecular protein complexes called focal adhesions, which are prerequisites for haptotaxis^12–14^. Ligand concentration differences between the leading and trailing edges determine the cell’s migration polarity^15^. When a cell is presented with a chemoattractive protein surface cue, the leading edge undergoes actin polymerization that triggers membrane protrusions and actin retrograde flow—key processes that drive cell migration^16–18^.

Studies of haptotactic signaling in vitro have monitored cellular responses to proteins patterned as either stripes or surface gradients. These surface-bound protein patterns can be formed using different techniques including microfluidic serial diluters and gradient generators, microfluidic probes, hydrogel stamp diffusion, dip-pen nanolithography, laser-assisted protein adsorption by photobleaching (LAPAP), porous capillary filters, and microcontact printing^19^. Stripe assays are widely used for haptotaxis experiments because they are relatively easy to generate and provide cell distribution results rapidly^20,21^. Ricoult et al.^21^, using classical stripe assays, demonstrated that haptotaxis is potently modulated by the cell-surface affinity of the reference surface (RS)—the area between the patterned protein cue—which in many cases dictated the cellular response. Haptotactic responses must therefore be interpreted in the context of the RS, yet the RS was often not considered in past studies or was not standardized, casting doubt on the validity of past results, and is expected to be a major source of irreproducibility. Ricoult et al. introduced an RS made of a mixture of poly-d-lysine (PDL, with cell adhesive properties) and polyethylene glycol (PEG, cell repellent), whereby the ratio of PDL and PEG was tuned to maximize cell response to the guidance cues^21^.

Classical stripe assays probe cell navigation only as a binary choice between a stripe without a cue, i.e., 0% coverage by the cue, and a stripe fully saturated with the cue, i.e., 100% coverage. Therefore, the classical stripe assay measures cell response only under the most extreme conditions, which is not representative of most in vivo conditions, and does not clarify the lowest coverage to which cells may respond, or how sensitive they are to relative changes in coverage. Gradient assays with a continuous range of density that can be produced with different slopes have been used to address some of the above shortcomings. Studies of the haptotactic response of rat hippocampal and epithelial cells on continuous gradients of laminin or fibronectin showed that cellular response is heightened on steep slopes, while higher fibronectin concentrations were associated with attenuated responses, potentially due to receptor saturation^22–24^. Digital gradients formed by either having a fixed unit pattern (i.e. a dot or a line) or having fixed spacing and varying pattern density afford quantitative gradient formation without uncertainty. Lang et al.^25^ and von Philipsborn et al.^26^ developed digital gradients composed of micrometer-scale dots or lines to study the repulsion of growth cones from temporal retinal ganglion cells towards ephrinA5. Our group introduced low-cost digital nanodot gradients (DNGs) to study cell migration^27^ and designed a set of 100 DNGs formed of 200 × 200 nm^2^ nanodots. DNGs with ordered and random nanodot distributions, with different ranges of linear and exponential slopes, and with different noise patterns were designed with a large dynamic range of up to 3.86 orders of magnitude^27,28^. These DNGs reflect the noncontinuity and large dynamic range characteristic of in vivo gradients^11^ and were used in cell migration studies^29^.

A study previously identified that for single ligands, RGD integrins ought to be no more than 60 nm apart to support cell adhesion, cell spreading and the formation of stable focal adhesions^12,30,31^. Ligand spacing of >73 nm led to erratic filopodia protrusions or even apoptosis, strongly suggesting that integrin nanoclustering is crucial for cell signaling. Coyer et al.^14^ found the minimal area of integrin−ligand complexes for fibronectin to be 333 × 333 nm^2^, suggesting that nanodots smaller than this threshold are not sufficient to support cell migration. However, others observed normal cell responses towards dots as small as 200 nm in diameter^32^. Haptotactic gradient designs reported in the literature are made of (nano)dots of varying sizes from 0.01 µm^2^ (100 nm × 100 nm) to ~12.25 µm^2^ (3.5 µm × 3.5 µm), but how dot size affects cell navigation was not thoroughly studied^26,27,33^. Considering the apparent contradictions regarding minimal dot size for focal adhesion formation, further investigation is needed to gain a better understanding of how nanodot size could affect haptotaxis.

Gradient haptotaxis assays using attractive cues afford a simple readout of cell response via the accumulation of cells at the high-density end of the gradient, but they suffer from a number of limitations. First, a gradient presents a defined slope and hence tests cell navigation only under this one condition. Second, cell accumulation after migration does not indicate where a particular cell was initially seeded on the gradient or where it came into contact if it entered from an edge; previous studies showed that fractional change, which for linear gradients is high only in the low-density zone, should be considered^34^. Third, cells navigating along a gradient are continuously exposed to the cue, which could lead to saturation. Fourth, many cells exhibit migration directional persistence, and hence cell accumulation may not be an accurate readout of cell choices, especially at the high end of the gradient^22,35^. Using arrays of gradients and live imaging could address some of these limitations, but the latter is much more cumbersome and does not resolve the issue that polarization, directional persistence, and different cell migratory histories may confound choices made by cells.

Herein, we introduce the digital nanodot stripe assay (NSA), which challenges cells with different discrete protein concentration steps, can span the entire surface coverage range, and probes different surface coverages using NSA combinations. Cell migration choices under multiple different conditions can thus be easily and quantitatively acquired in a single experiment, without interference from cell navigation history or directional persistence. Sixty-three NSAs with nanodot arrays with two different surface coverages in a stripe pattern were designed. Coverages including the two extremes of 0 and 100% as well as five intermediate values, and three different nanodot sizes were tested. To maximize and standardize the cell response, a RS made of an optimized ratio of PLL:PEG was used. All NSAs were patterned on an Si wafer that was used as a master for creating PDMS stamps for nanocontact printing. Netrin-1 was then patterned on a surface by lift-off nanocontact printing. To probe cell choices, we studied the haptotaxis response of C2C12 myoblasts towards the widely studied neuronal guidance cue netrin-1^36^. C2C12 cells expressed neogenin, a transmembrane receptor for netrin-1^37^, which has been verified to trigger chemoattractive haptotaxis responses when bound to netrin-1^21^. C2C12 myoblasts were seeded on all NSAs simultaneously, and the cell distribution—reflecting the cell choice—was recorded at the end of each experiment for all 63 combinations at once. The cell accumulation for each combination was analyzed, and the response was further compared to one obtained with a step gradient formed using identical stripe arrays arranged in order of increasing density.

## Results and discussion

### NSA design and fabrication

**Fig. 1:**
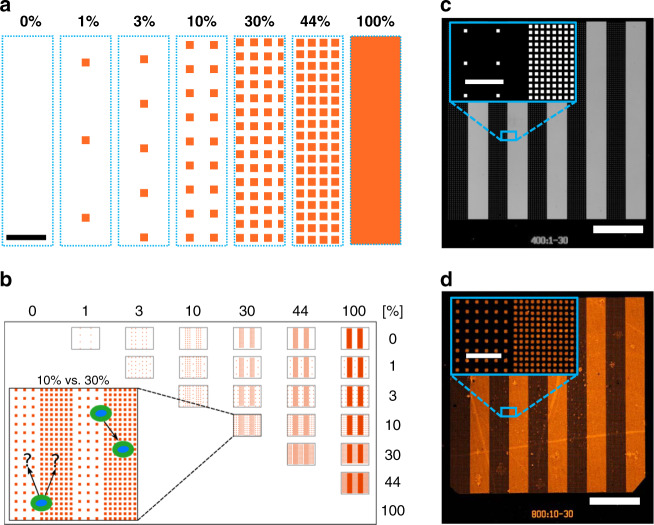
Nanodot stripe assay (NSA) combinations. **a** Schematic visualizations of the seven different nanodot stripe arrays with different surface coverages tested here. The arrays represent a small cutout area of a stripe representing the actual surface coverage proportions. **b** Schematic overview of all 21 NSA combinations designed and used to test cell choice across a range of absolute and differential densities in a single experiment. The density for the high- and low-density stripes are shown on the top and right axis, respectively. The inset illustrates cells seeded on the NSA making a choice between stripes with 10% vs. 30% protein density. A single NSA covers an area of 400 × 400 µm^2^ and includes five pairs of adjacent stripes, each 40 × 400 µm^2^ in size and formed by an array of nanodots. **c** An NSA made of 400 × 400 nm^2^ nanodots with 1% and 30% surface coverage stripes, respectively (labeled as 400:1−30), etched into Si. Inset shows the boundary between two stripes. **d** 800:10–30 NSA (with 10 and 30% stripe coverage) of surface-immobilized fluorescent netrin-1 proteins patterned as nanodots by nanocontact printing. Inset shows the boundary. Scale bars: **a** 1 µm, **c** 100 µm, **d** 100 µm (10 µm for inserts).

The NSA we introduce consists of stripes of ordered nanodot arrays patterned in a square lattice with seven surface coverages of 0, 1, 3, 10, 30, 44 and 100% (Fig. 1a). These seven surface coverages are patterned side-by-side as stripes according to all possible 21 pairwise combinations (Fig. 1b and Supplementary Table S1). Each stripe measures 40 µm × 400 µm, and five pairs of alternating stripes with a lower and a higher density from an NSA with a 400 × 400 µm^2^ footprint (Fig. 1c, d). The density increase factor was arbitrarily chosen to be 3 and 3.33 as a trade-off between minimizing the smallest multiplicative increase on one hand, and avoiding a large number of combinations and increased manufacturing costs on the other hand. There were two exceptions dictated by two surface coverage densities: (i) The inclusion of 0%, which leads to an infinite multiplicative factor to all other surface coverages, and (ii) the inclusion of a 44% density stripe because 44% is the highest bona fide nanodot array density that could be made with 200 × 200 nm^2^ nanodots. At densities above 44%, individual nanodots could not be fabricated without compromising the structural integrity of the nanostructures. The inclusion of the 44% density added a ratio of 1.47 and 2.22 relative to 30 and 100% coverages, respectively.

To test the effect of nanodot size on cell migration, we designed the NSA with arrays of three different nanodot dimensions: (i) 200 × 200 nm^2^, (ii) 400 × 400 nm^2^ and (iii) 800 × 800 nm^2^. The same nanodot size was used for each NSA, and the density of dots on the stripe arrays was adjusted accordingly, i.e., cell choices were always between two stripes with the same nanodot size; 0 and 100% were again exceptions, as they do not include nanodots per se. Accordingly, we designed three sets of 21 unique binary combinations, totaling 63 nanodot stripe pairs per print and per combinatorial NSA experiment. To enable the study of migration across the different surface densities, a step gradient made of five nanodot arrays with 1, 3, 10, 30 and 44% coverage was added to the 21 NSA combinations (one for each nanodot size, i.e. 3 in total), forming staircased surface patterns. The final design layout, with all 63 combinatorial NSAs and the 3 step gradients, is shown in Supplementary Fig. S1. Whereas we previously showed that protein nanodots as small as 100 × 100 nm^2^ could be patterned^27^, 200 × 200 nm^2^ was the smallest nanodot size used here because it represents the lower limit that allows both repetitive and reliable nanocontact printing, which is essential for initial exploration, optimization, and the production of the large number of prints needed to perform replicate cell migration experiments.

The stripe width was chosen to strike a balance between minimizing the population of cells that would either be (i) positioned only on one-density stripe or (ii) extended across a full stripe onto both adjacent stripes on the left and right. If stripes are too wide, then many cells seeded on the stripe array are at risk of experiencing only one surface coverage, as described in scenario (i), and provide no useful information about cell migration choices. If the stripes are too narrow, then both the leading and trailing edges of cells can extend to adjacent stripes with the same protein concentration, as described in case (ii), and thus prevent the cell from making a clear choice. Based on the typical size of C2C12 myoblasts that are approximately 20 µm immediately following sedimentation, which then spread out further upon adhesion, a stripe width of 40 µm was chosen.

The fabrication of the nanodot arrays and the deep trenches for 100% coverage in a silicon (Si) wafer was achieved using cleanroom nano- and microfabrication techniques including electron beam lithography, standard lithography and etching processes. The combinatorial NSA fabrication process flow is shown in Supplementary Fig. S2 with the resulting Si patterns illustrated in Supplementary Fig. S3. The replication of Si patterns onto PDMS stamps and the surface patterning of nanodots of proteins by lift-off nanocontact printing are described in detail in the “Methods” section and illustrated in Supplementary Fig. S4. Both 0 and 100% coverage do not require nanopatterns per se. NSA are labeled as C:A–B, where C is the nanodot size, A is the lower-density stripe array, and B is the higher-density stripe array. For example, 200:3–30 refers to a NSA with 200 × 200 nm^2^ nanodot size with alternating nanodot stripe arrays with 3 and 30% coverages. The NSA design thus resembles a classical stripe assay format, but made of nanodots of different surface coverages to modulate the local surface protein density, and includes intermediate coverages within the extremes of 0 and 100% coverages. In summary, using a combination of 21 NSAs, the haptotactic response of cells to 21 different nanodot stripe pairs, ranging from low to high surface densities and small to large steps could be quantified in a single experiment as shown in Fig. 1 and Supplementary Figs. S1 and S3.

### Cell migration choices on netrin-1 NSA

**Fig. 2:**
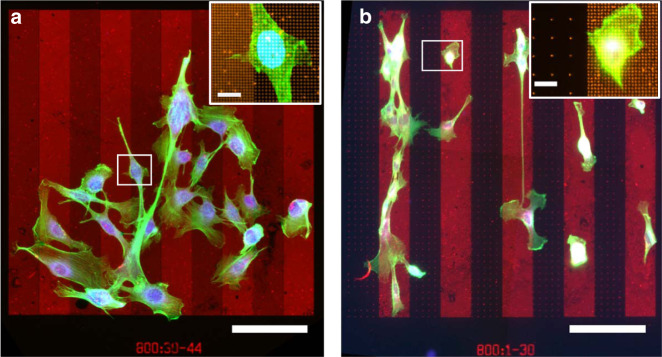
Fluorescent micrographs of C2C12 myoblasts on stripes of surface-bound netrin-1 reveals haptotaxis. Composite image of netrin-1 (red), cells (green) and nucleus (blue) reveals the distribution of cells on stripe arrays with the experimental conditions: **a** 800:30–44 and **b** 800:1–30. Although no cell preference is apparent in (**a**), cells are preferentially positioned on the higher-density stripes in (**b**) as a result of haptotaxis. The RS between the dots was backfilled with 90:10 %PEG:%PDL. Scale bar is 100 µm and 10 µm for insets.

In this study, we used combinations of NSAs to examine contact-mediated haptotaxis choices of C2C12 myoblasts towards different density nanodot stripes of netrin-1. C2C12 cells scan the contiguous microenvironment, resulting in receptors binding to netrin-1 anchored on the surface. Based on the surface ligand density, C2C12 myoblasts establish a migration polarity that may cause cells to preferentially migrate to an adjacent nanodot stripe, eventually accumulating on the stripe with the preferred surface protein concentration. Figure 2 shows migrated myoblasts on fluorescent netrin-1 prints at the end of the migration assay. Cell choices per NSA were quantified as the percentage of cells on the higher-density stripe in relation to the total number of cells (Supplementary Fig. S5). To confirm that cellular haptotaxis choices were not made by chance, we compared migration results on netrin-1 NSA’s to negative control assays, where we printed fluorescent immunoglobulin-G (IgG) as a substrate control.

### Cell migration choices are independent of protein nanodot size between 200 and 800 nm

**Fig. 3:**
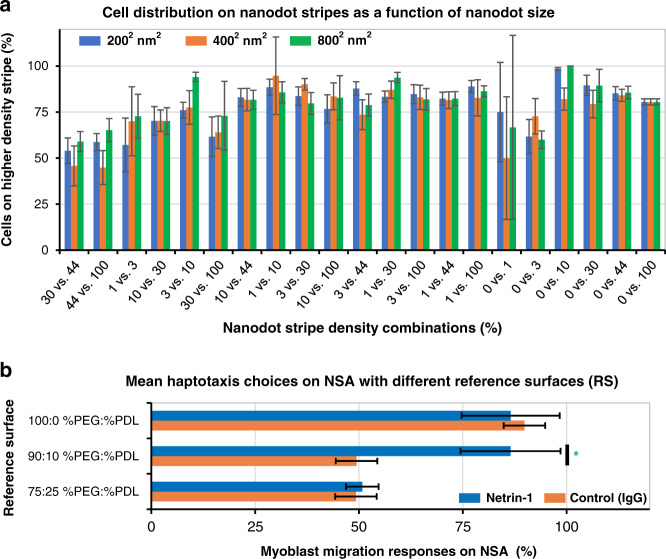
Cell migration preferences are independent of protein cluster sizes but sensitive to the cell-surface affinity dictated by the reference surface. **a** Cell choice results on netrin-1 NSAs are insensitive to nanodot size for dimensions of 200 × 200 nm^2^, 400 × 400 nm^2^, and 800 × 800 nm^2^. Random distribution of cells on the stripes would result in ~50% on the higher-density stripes, and many conditions reveal a higher fraction of cells on the higher-density stripes. However, nanodot size does not markedly affect cell distribution; all experiments were conducted with the optimal RS, and subsequent analyses were conducted by combining results obtained on arrays with same density but different nanodot size (see below for more details). Low numbers of adherent cells on stripes with 1 and 3% coverage account for larger error bars. Error bars indicate the standard error of the mean (SEM). **b** A summary of migration choices across different RSs, showing specific cell migration only for the 90:10 %PEG:%PDL RS. For the low cell-surface affinity with 100% PEG, RS cells migrate onto stripes with a higher-density of cues whether patterned with netrin-1 or IgG. Conversely, on RSs with the high cell-surface affinity associated with 75:25 %PEG:%PDL, cells are randomly distributed irrespective of netrin-1 or IgG surface coverage. Asterisk (*) indicates statistical significance, where *p* = 3.1 × 10^−11^. Error bars indicate the standard error of the mean (SEM).

We examined the effects of nanodot size on cell migration choices by observing the haptotactic responses of myoblasts on combinatorial NSAs of netrin-1 made with each of the three different nanodot sizes. We verified that our printed nanodots matched the overall design, and the efficacy of the lift-off nanocontact printing method used here had previously been confirmed^27,38^. We observed that cell choices were independent of the size of the protein nanodots and that netrin-1 nanodots from 800 × 800 nm^2^ (0.64 µm^2^) down to 200 × 200 nm^2^ (0.04 µm^2^) elicit the same level of response (Fig. 3a). These results appear to contradict some previous publications^12–14,30,33,39^ and are not consistent with the observations made by Coyer et al., who reported that 333 × 333 nm^2^ = 0.11 µm^2^ fibronectin nanoislands were not sufficient for focal adhesions maturation^14^. However, it should be noted that the specific ligand being patterned, the surrounding RS (discussed further below), the protein density and the particular cell type are not consistent among these studies. These unique attributes of the various assays are likely to account for the differences observed.

### Identifying the optimal reference surface

As discussed in the introduction, controlling the cell-surface affinity of the RS is critical, as it was previously shown that for high or low affinity RSs, observed haptotactic responses may be strongly driven by attraction or repulsion to the RS rather than the cue of interest^21^. In this study, we tested cell choices on substrates patterned with netrin-1 or IgG (control) NSAs, backfilled with three different concentration ratios of PEG and PDL (%PEG:%PDL) to modulate the cell-surface affinity of the RS. We first evaluated cell adhesion and growth on RSs (without any patterns) with ratios between 100:0 and 75:25 %PEG:%PLL (Supplementary Fig. S6). For surfaces with 100 and 95% PEG, the adhesion was significantly reduced, while for ratios of 10 and 25% PEG, myoblast adhesion and growth were indistinguishable from those on surfaces homogeneously coated with PDL or netrin-1. Hence, for surfaces with at least 10% PDL (and at most 90% of PEG), cell adhesion was preserved, which is a slightly higher threshold than that observed in our previous experiments^21^.

In a first haptotaxis experiment, NSAs with an RS composed of 100:0 %PEG:%PDL, produced by backfilling the nanopatterned substrate with 10 µg/mL PEG, were used for cell choice assays. C2C12 myoblasts were found to migrate preferentially onto nanodot stripes with higher protein surface densities, irrespective of whether they were functionalized with netrin-1 or IgG, indicating that cellular responses were dominated by repulsion to the RS, as expected (Fig. 3b and Supplementary Fig. S7-A). Conversely, at a higher cell-surface affinity, the RS prevented cells from migrating toward higher densities of netrin-1. Indeed, for an RS of 75:25 %PEG:%PDL, cells on netrin-1 or IgG NSAs were randomly distributed, and no cellular choices favoring higher-density arrays were observed (Fig. 3b and Supplementary Fig. S7B). Substrates coated with high PDL concentrations have been shown to slow the velocity of cell migration due to the high level of relatively nonspecific cell-substrate adhesion^21^. These three different RS affinities resulted in markedly different cell migration choices with two cases highlighting the capacity of the RS to interfere with chemoattractant directed cellular migration.

### Cells preferentially choose higher netrin-1 density stripes

**Fig. 4:**
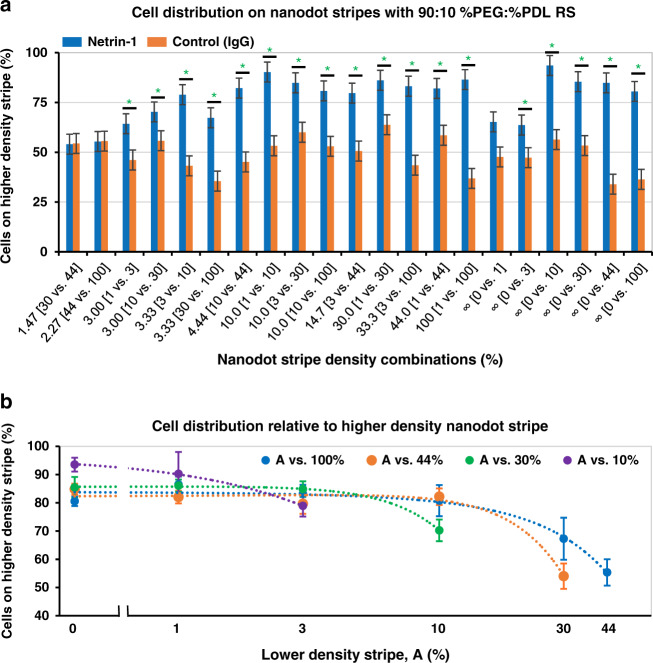
NSAs quantify C2C12 myoblasts haptotaxis to areas with higher netrin-1 coverage and reveal the dependency of surface ratio and average coverage of stripes. **a** Cellular responses to NSAs show increased response with increasing ratio of high- to low-density stripe (B/A) for netrin-1 but no response for NSAs with IgG nanodots (negative control), both with an RS of 90:10 %PEG:%PDL. Asterisks (*) mark NSAs with statistical differences where *p* < 0.05 between netrin-1 and IgG control (see Table S2 for details). **b** Cell distribution plots of haptotaxis choices to netrin-1 NSAs with constant high-density stripe versus increasing lower-density stripe; A vs. 3% and A vs. 1% are not shown because of low cell counts. Cell preference for a higher-density stripe decreases as the fold-change between the two nanodot stripes decreases below ~4; the dotted lines are visual guides. Error bars indicate the SEM.

For an RS coated with 90:10 %PEG:%PDL, C2C12 myoblasts migrated preferentially onto higher netrin-1 density nanodot stripes for 18 out of the 21 (~85%) NSA subarrays (Fig. 4a). Nanodot stripes of the IgG negative control patterned on this RS resulted in random cell distributions, confirming that this RS results in netrin-1-specific cellular responses. As discussed previously, the response was insensitive to nanodot size for the range studied here (Fig. 3 and Supplementary Fig. S8). The NSA cell choice results in Figs. 3a and 4a are arranged in order of increasing ratio of protein density fold-change between the two alternating stripes calculated by dividing the density of the higher with the lower stripe. For all pairs with a fold-change >3.33, as well as the pairs including a zero-density stripe without nanodots (∞ ratio), ~80% or more cells were located on the higher-density stripe, with the exception of stripe pairs 0 vs. 1% and 0 vs. 3%. These results suggest that a ratio of 3.33 or higher and nanodot density >3% (situated between 3 and 10%) are needed to elicit the maximal cell response for C2C12 cells and netrin-1 nanodots. The low response detected on low-density stripes can be accounted for by single cells being in contact with only a few nanodots. For instance, stripes made of 200 × 200 nm^2^ dots at the 1 and 3% densities have nanodots with center-to-center spacing of 2 µm and 1156 nm (scaling to 4 µm and 2.312 µm for 400 × 400 nm^2^ dots). A hypothetical cell on a 1 or 3% stripe occupying a 10 µm square would contact only ~25 or ~100 nanodots, respectively.

Cells were subjected to a surface coverage ratio of 3 or 3.33 in four different stripe pairs: 1 vs. 3% and 10 vs. 30% for a ratio of 3 and 3 vs. 10% and 30 vs.100% for a ratio of 3.33. The cell preference for the higher-density stripe was 65−67%, except for 3 vs. 10%, which yielded a cell choice of 79% on the 10% stripe, similar to the cell choice observed for higher-coverage ratio (4.4 and greater in this study). Only two conditions with ratios <3 were tested thanks to the inclusion of a stripe with 44% density. Interestingly, neither for 30 vs. 44% (1.47) nor for 44 vs. 100% (2.27) did we observe a cell choice. Hence, in the experimental conditions tested, a ratio of 3 or higher was needed to elicit a cellular response. The cell choice observed for 30 vs. 100% indicates that saturation alone cannot explain the lack of response in both cases. However, saturation could contribute to attenuating the response, as the cell choice for 30 vs. 100% was less pronounced than for 3 vs. 10% or 10 vs. 30%.

Figure 4b showcases migratory cell choices for a specific high-density stripe vs. each of the different lower-density stripes. The choice is constant for most pairs but consistently drops off towards the end of the curve where the lower-density stripe approaches the surface coverage of the higher-density stripe, as one might expect. The cell choice is consistently at ~80% cells on the higher-density stripes, with the exception of the series of experiments with a 10% high-density stripe, which show a cell preference reaching >90%. Additional experiments may be required to confirm this trend and could be completed by experiments that test for cell signaling and inhibition to reveal additional facets of cell haptotaxis.

In summary, a ratio of at least ~2.3 was needed to elicit a cellular choice. In this assay, maximal cell preference for higher-density stripes plateaued at ~80% of cells residing on the higher one, and this plateau was reached for density ratios between 3.33 and 4.44, while for these relatively low ratios, cellular choices are less pronounced at the extremes (low and high) of surface coverage.

### Cell migration on a step gradient made of stripes of nanodot arrays

**Fig. 5:**
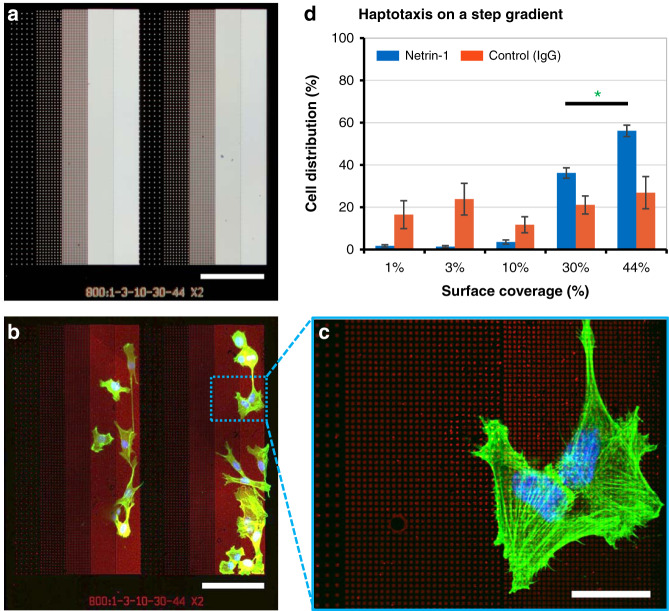
Cell haptotaxis on step gradients formed by nanodot stripe arrays reveals a cell distribution consistent with migratory persistence. **a** Step gradient on a Si mold showing 1, 3, 10, 30 and 44% nanodot stripe arrays, repeated twice in the *x*-direction and made of 800 × 800nm^2^ nanodots. **b** A composite image of myoblasts on the step gradient showing cells that preferentially migrated onto the 30 and 44% density stripes. **c** An inset showing a close-up view of C2C12 myoblasts on netrin-1 nanodots. **d** Cell distribution on the step gradient reveals cell accumulation towards the high coverage of the gradient of netrin-1, with substantially higher numbers on the 44% vs. 30% stripes, which is inconsistent with the absence of choice observed on the NSA. Error bars indicate the SEM. Scale bar is 100 µm and 20 µm for inset. * indicates *p* = 7.8 × 10^−5^.

The haptotaxis results obtained with the NSAs were compared to a step gradient formed using identical stripes to the ones used in the NSAs but arranged in order of increasing surface coverage: 1, 3, 10, 30 and 44%. We note that in this design, cells could migrate to the left from the central 1% stripe directly onto the 44% stripe; however, because the number of cells adhering on 1% stripes was very low, we expect the number of cells migrating in this way to be insignificant. A robust directional migratory response to the gradient was observed (Fig. 5). As expected, C2C12 myoblasts migrated in the direction from lower-density to higher-density stripes of netrin-1 coverage. Approximately 6% of the C2C12 myoblasts preferred arrays with 1, 3 and 10%, with the majority (~94%) migrating to the stripes with 30 and 44% coverages. Interestingly, ~20% more cells were detected on the stripe with a coverage of 44% compared to the ones with 30%, which is consistent with other gradient assays^27^. These results are inconsistent with the binary choice assays within the NSA that found no difference in cell accumulation between 30 and 44% coverages (see Fig. 4). The accumulation of cells on the higher-density stripes is however consistent with directional persistence of cell migration. Directional persistence here means that motile cells seeded on the lower-density stripe arrays (1, 3, and 10%) continued their migration past the 30% stripe into the 44% stripes simply by maintaining their migration direction. Long timescales of directional persistence have been reported to occur in stimulated cells, leading to directional persistence times on the order of minutes^40^. Signaling pathways involving the small GTPase Rac and Arp2/3 are known to prolong positive feedback loops that maintain actin polymerization at the cell’s leading edge and sustain the cell’s direction based on initial (preceding) events^35^. Our results suggest that the cell distribution on the step gradient is confounded by migratory cell persistence and hence reflect the integration of cell choices and cell history via directional persistence. We foresee that the results of the step gradients would be replicated on continuous gradients, but experimental confirmation is not available at this time. Nonetheless, the combined results of the NSAs and the step gradient assays indicate that end-point measurements in gradient-type experiments cannot distinguish between cell choice and persistence due to migratory history.

## Conclusions

A combinatorial array of NSAs was used to systematically study cellular haptotactic choices between lower and higher protein surface densities across seven different coverages and three nanodot sizes resulting in 63 different binary concentration tests. The NSA simultaneously probes local sensitivity, the minimum surface ligand concentration required to trigger haptotactic responses, the effect of absolute coverage and coverage ratio, and the saturation of haptotactic responses by adherent cells. For C2C12 myoblasts expressing neogenin and exposed to NSAs with netrin-1 nanodots on a RS surface with 90:10 %PEG:%PDL on 40-µm-wide stripes, the following response was observed: (i) the minimum cell-recognizable netrin-1 surface concentration is ~3% coverage; (ii) a protein concentration coverage fold-change of >2.3 across a cell’s leading and trailing edges is needed to elicit a choice; (iii) cell choice saturates at 80−90% of cells migrating to the higher-coverage surfaces for ratios >3.3 (for 10% coverage) or >4 (for coverage below and above 10%). The NSAs identify the thresholds and responses of cellular choice that are not accessible to classical 0% vs. 100% stripe assays or in vivo experiments. These results are pertinent to the dynamic range of netrin-1 gradients observed in chick embryos^11^. Our results revealed a discrepancy between cell choice on the NSA, which showed no choice between 30 and 44% coverage but a greater accumulation of cells on the 44% surface in a step gradient. These results are consistent with cell migration persistence leading to accumulation at the top of the gradient, and hence cell choice on gradients integrates the results of cell choice and directional persistence. These results need to be confirmed on continuous gradients, and further insights could be gained by live cell imaging to confirm and provide stronger evidence of migratory persistence dictating the final cell position. In vivo patterns often follow a gradient shape, and hence a gradient remains a physiologically relevant test; however, the final position of the cells on the gradient cannot be used to conclude that cells are able to respond to high-density gradients. Conversely, by combining NSAs and gradients, the migratory persistence of cells could be made apparent.

We found that cell choices were independent of the size of protein nanodots from 200 × 200 nm^2^ (0.04 µm^2^) to 800 × 800 nm^2^ (0.64 µm^2^), where each nanodot consists of a multitude of netrin-1 ligands, and which mediate ligand cluster dimensions. We conclude that for the range studied, cell haptotaxis decisions are dictated by the overall protein surface concentrations experienced by the entire cell. The ultimate limit for nanodot size is single-protein nanodots, and further experiments are needed to determine whether for a fixed protein density, there is a threshold for cell response based on a minimal size, or whether nanodot order and distribution could have an effect^28,29^. Quantitative molecular data from nanodot arrays could be obtained using live cell microscopy, which could be used to quantify focal adhesion size across the different nanodot sizes along with cell migration velocities and thus contribute to unraveling the relationship between nanodot size and focal adhesion formation. Our results complement other studies addressing the interplay of ligand cluster sizes and protein (ligand) distributions^30,41^. Future experiments using low-cost and high-throughput NSAs could include quantitative focal adhesion dynamics using advanced fluorescent microscopy methods^13,42,43^ and integrate receptor inhibition or sensitization to further explore signaling and saturation in various combinatorial scenarios. In our study addressing the functionality of the reference surface^21^, markedly different cell-surface affinity curves were observed for different cell types and cues, and the interplay between guidance cues, including extracellular matrix proteins like fibronectin, laminin and vitronectin, neuronal guidance cues like slits, ephrins and semaphorins, the migratory cell type, and the RS, are expected to result in different quantitative cell choice results on combinatorial NSAs. NSAs could be further expanded to test chemo-repellent cues, to include soft substrates as previously introduced by our group^36^ and others^44^, and to examine the interaction between different cell types. The knowledge gained from NSAs could help understand cell choice in the context of tissue repair as well as to help design implants that efficiently guide neurons to electrical contacts for improved device-to-body interfaces^45^.

Our results underline the value of in vitro experiments to expose and quantify the relationships between specific parameters and cell migration thanks to systematic variation of one parameter at a time. This experimental simplicity is typically not accessible for in vivo studies because many parameters are linked and codependent, and it is very difficult to distinguish between direct and indirect effects. The results of in vitro studies on 2D surfaces are not directly translatable to in vivo, but the results can guide the design and interpretation of in vivo studies in tissues and embryos, thanks to the phenomena revealed and the quantitative relationships determined.

## Materials and methods

### NSA design and Si mold fabrication

Arrays of square nanodots with two different densities were patterned as a series of ten contiguous 40 × 400 µm^2^ stripes using L-edit from Tanner design tools. Design files from L-edit were exported to .gds files in preparation for the fabrication process. Stripe combinations are summarized in Supplementary Table S1 followed by a mask layout shown in Supplementary Fig. S1. The arrays were patterned onto a silicon (Si) wafer at the Institut National de la Recherche Scientifique (INRS) using electron beam lithography (EBL) and direct UV laser writing for the nano- and microsized features, respectively (Supplementary Fig. S2). In brief, a two-step lithography process was employed to (1) pattern nanoscale features (i.e. the nanodots), and to (2) pattern microscopic features, such as the 40 µm × 400 µm stripes with 100% coverage. The Si wafer was first spin-coated with a 200 nm layer of positive ZEP520a resist. Nanodots were then patterned using EBL onto the ZEP520a resist layer. After development, the Si was dry etched 200 nm deep using a SF_6_/C_4_F_8_ plasma. Prior to fabricating the 100% coverage stripes, the wafer was spin-coated with positive Shipley 1813 resist, resulting in a 2-µm-thick coating. The mask was aligned to the wafer with the nanodots using fiducial markers and patterned via direct UV laser writing. Nanodot stripes and 100% stripes were designed to overlap slightly to account for misalignment of up to 4 µm in the horizontal and vertical directions. Finally, deep reactive ion etching into Si using the Bosch process created 5 µm deep trenches. The remaining photoresist was stripped away to complete the fabrication process. Supplementary Fig. S3 shows a collage of the final patterns on Si.

### PDMS and NOA mold replication

The low-cost lift-off nanocontact printing procedure introduced by Ricoult et al.^27^ was utilized to generate secondary molds and to pattern netrin-1 nanodot stripes. The process steps are summarized in Supplementary Fig. S4. Briefly, the Si mold was replicated first into a polydimethylsiloxane (PDMS) elastomer and then into a UV-sensitive polyurethane replica for lift-off printing of proteins adsorbed on a flat PDMS stamp. First, the Si mold was coated with trichloro(1H,1H,2H,2H-perfluorooctyl)silane (Sigma-Aldrich, Oakville, ON, Canada) via vapor deposition to prevent PDMS sticking. PDMS base, Sylgard 184 (Dow Corning, Midland, Michigan, USA), was mixed with its curing agent at 10:1 (v/v), degassed, and cured on Si in an oven (VWR, Montreal, QC, Canada) at 60 °C for at least 12 h. To prevent PDMS leaching, uncured oligomers were extracted by submerging PDMS in 70% ethanol overnight, then dried at 60 °C for 6 h. A UV-curable NOA-63 (Norland Optical Adhesive, Norland Products, Cranberry, NJ, USA) stamp was then replicated from the PDMS mold. NOA-63 stamps had identical features to the original Si mold.

### Protein patterning

To print proteins (Supplementary Fig. S4), ~1 × 1 cm^2^ planar PDMS stamps were sonicated in 70% ethanol for 30 min and dried using a strong nitrogen stream. Thereafter, each stamp was inked with 15 µL of netrin-1 at 25 µg/mL, covered with a cover slip to evenly spread the protein solution, and incubated for 10 min. Fluorescent goat-anti-rabbit immunoglobulin-G (IgG) conjugated with Alexa Fluor 546 (Invitrogen, Burlington, ON, Canada) was mixed with netrin-1 at 4:1 concentration ratios to visualize printed spots. The stamp was then dried under a stream of nitrogen and placed in contact with a plasma-treated (PlasmaEtch PE-50, Carson City, NV, USA) NOA stamp to lift-off the unwanted proteins. The residual protein pattern on the PDMS stamp was then transferred to a plasma-treated (1 min) cover slip by printing it and leaving it in (conformational) contact for 10 s. Cover slips with netrin-1 prints were quickly placed in well plates, backfilled with the desired RS and incubated in a cold room for 1 h.

### Reference surface (RS) solution preparation

The RS solutions were made by mixing a copolymer, poly(l-lysine) grafted with poly(ethylene glycol), PLL(20)-g[3.5]-PEG(2) (Surface Solutions, Dubendorf, Switzerland), abbreviated PEG, with poly-d-lysine (PDL, 70−150 kDa, Sigma-Aldrich, St. Louis, MO, USA) at the desired concentration ratios. PDL is a synthetic, enzyme-resistant D-amino-acid analog of polylysine. PEG has a very low cell-surface affinity, while PDL has a very high cell-surface affinity. Stocks of PEG and PDL were diluted in phosphate-buffered saline (PBS) to 10 µg/mL prior to mixing at the desired volume ratios. RS incubation lasted 1 h and was followed by aspiration using a pipette and rinsing (3×) with 1× PBS.

### Cell culture procedures

C2C12 cells (ATCC, Manassas, VA, USA), a mouse myoblast muscle cell line that expresses neogenin, a transmembrane receptor for netrin-1 and a DCC paralogue^37^, were cultured following standard cell culture procedures. Briefly, cells were cultured in high glucose DMEM (Dulbecco’s modified medium), supplemented with 10% fetal bovine serum and 5% penicillin/streptomycin (all from Invitrogen, Burlington, ON, Canada). Cells were passaged every 2–3 days, re-seeded at 10^5^ cells per 25 cm^2^ cell culture flask, and kept in an incubator at 37 °C with 5% carbon dioxide. A fraction of the passaged cells was seeded onto wells with cover slips patterned with netrin-1 nanodot stripes (or IgG) with the desired RS at 2.8 × 10^3^ cells/cm^2^—estimated from hemocytometer counts.

### Cell staining and imaging

After 18 h, the cultured cells were fixed with 4% paraformaldehyde and 0.2% (v/v) glutaraldehyde (Sigma-Aldrich, Oakville, ON, Canada) for 4 min. The cells were then permeabilized with 0.15% Triton X-100 in PBS for 4 min, and blocked with 3% Horse Serum for at least 1 h. Thereafter, cells were stained for 30 min with Hoechst (1:10,000) and phalloidin conjugated with Alexa Fluor 488 (1:250) to label the nucleus and actin filaments, respectively (all from Invitrogen, Burlington, ON, Canada), rinsed (3×) with 1×-PBS, and imaged with a fluorescence microscope (TE2000, Nikon). A multiposition and multichannel NIS Elements script was used to capture images for the netrin-1 patterns and cells. Images were overlaid to determine the location of cells on the netrin-1 nanopatterns with the center of the cell’s nucleus taken as the cell’s centroid.

### Data extraction and analysis

ImageJ (Fiji) was used to overlay fluorescent images of the netrin-1 prints and cells. A region of interest (ROI) was selected based on the boundaries of the netrin-1 print, and the particle analysis module was used to find cell locations relative to the netrin-1 patterns (Supplementary Fig. S5). A MATLAB script was developed to identify which design and density arrays the cells were located on. Each design consisted of two alternating stripes of nanodots arrays, i.e., array A and array B; therefore, cells could be on either A or B. This configuration reduces the cell migration test to an “ON” or “OFF” binary test, specifically, on B or off B. Myoblasts haptotaxis choice results were analyzed for each design by calculating the ratio of cells on each of the alternated arrays relative to the total cells. We calculated the relative cell distribution percentage using Eq. 1,

1 $${\mathrm{Cell}}\,{\mathrm{preference}} = \frac{{{\mathrm{number}}\,{\mathrm{of}}\,{\mathrm{cells}}\,{\mathrm{on}}\,B}}{{{\mathrm{number}}\,{\mathrm{of}}\,{\mathrm{cells}}\,{\mathrm{on}}\,A + B}},$$

where *A* and *B* are the coverage densities of the alternating arrays per design, and *B* has a higher-density than *A*. For instance, in the design 800:3–100, *A* = 3% coverage and *B* = 100% coverage. Statistical analysis to produce *p* values was obtained from two-tailed *t* test analyses comparing the ratio of cells on netrin-1 vs. IgG controls of the same stripe combination. Error bars were calculated using the standard error of the mean (SEM).

## Supplementary information

Editorial Summary

Supplementary Information

## Data Availability

The raw and processed data required to reproduce these findings are available upon request.
