# Supplementary material for: Combinatorial nanodot stripe assay to systematically study cell haptotaxis
Source: Microsyst Nanoeng. 2020 Dec 14;6:114. doi: 10.1038/s41378-020-00223-0 (PMC7735170; doi:10.1038/s41378-020-00223-0)
Supplement: Supplementary file 1 — Editorial Summary [file 41378_2020_223_MOESM1_ESM.docx]

# *Microsystems & Nanoengineering*

Cytology: Nanodot stripe assay for studying cell haptotaxis

Critical to cell guidance and development, haptotaxis is directional cell movement in response to an adhesive substrate, and a method has been developed using nanodot stripe assays (NSAs) to systematically study haptotaxis that supersedes conventional stripe assays. Hitherto, haptotaxis has been studied in vitro using standard stripe assays, which offer only a binary choice between full or zero response to a protein: the assays measure cellular responses under extreme conditions that do not reflect in vivo situations. However, a group headed by David Juncker at McGill University, Canada has succeeded in producing NSAs, which are formed by adjacent stripes of nanodot arrays, that in addition to the extremes, include 5 intermediate surface densities. The team used a combination of 21 NSAs of the Netrin-1 protein to study the migration choices of cells to both the extreme and 20 intermediate conditions. The authors believe their approach can better elucidate haptotactic choices and mechanisms.

Related article manuscript number: MICRONANO-01364R

Article title: Combinatorial nanodot stripe assay to systematically study cell haptotaxis

Corresponding author and affiliation/s: David Juncker, McGill University, Biomedical Engineering Department, Montreal, Quebec, Canada

**About your Editorial Summary — please read**

**Before approving this Editorial Summary, please carefully check that (1) the summary text lists the correct author(s) and (2) the spelling and order of all author names and affiliations are correct.**

This **Editorial Summary** is based on your manuscript that was recently accepted for publication in *Microsystems & Nanoengineering*. It provides a non-specialist audience with a synopsis of your key research outcomes and conclusions. This value-added service provided by Springer Nature is designed to raise interest in your research across the broader community.

Springer Nature will publish the summary on the journal’s website, and it will be freely available under a under the CC BY licence (Creative Commons Attribution v4.0 International Licence) (see the journal website for details). We encourage you to re-use the summary to bring attention to your research; for example, you can host it on your own website and share it via social-networking platforms. Please attribute the summary to *Microsystems & Nanoengineering* and your article (e.g. by providing a link to your article) and do not make derivatives.

Please note that to maximise the usefulness of these summaries they must follow several stringent guidelines:
-- Spelling, punctuation and style are set according to *Nature* editorial guidelines. As this summary is aimed at non-expert readers, some concepts and technical terms will be simplified.
-- Total length must be no more than 135 words. It is likely that not all points in the paper will be covered.
-- The first sentence must be no more than 280 characters, including spaces, to allow use on microblogging sites.
-- The headline must consist of a brief generic subject identifier followed by a short description. No more than 10 words in total.

Please contact the editorial office ([mems_nano@mail.ie.ac.cn](mailto:mems_nano@mail.ie.ac.cn)) immediately with corrections should you find any factual errors in this Editorial Summary.
