# Supplementary material for: Combinatorial nanodot stripe assay to systematically study cell haptotaxis
Source: Microsyst Nanoeng. 2020 Dec 14;6:114. doi: 10.1038/s41378-020-00223-0 (PMC7735170; doi:10.1038/s41378-020-00223-0)
Supplement: Supplementary file 2 — Supplementary Information [file 41378_2020_223_MOESM2_ESM.docx]

# Supplementary Information

**COMBINATORIAL NANODOT STRIPE ASSAY TO SYSTEMATICALLY STUDY CELL HAPTOTAXIS**

Mcolisi Dlamini^1,2,3^, Timothy E. Kennedy^3,4^ and David Juncker^1,2,3,4*^

^1^Biological and Biomedical Engineering Department, McGill University, Montréal, QC, Canada

^2^McGill Génome Centre, Montréal, QC, Canada

^3^McGill Program in Neuroengineering, Montréal, QC, Canada

^4^Department of Neurology and Neurosurgery, McGill University, Montréal, QC, Canada

*email address: [david.juncker@mcgill.ca](mailto:david.juncker@mcgill.ca)

## NSA coverage combinations and design equations

The number of discrete coverage densities for the NSAs was limited to seven between 0% and 100% inclusive. A 100% array is a stripe pattern, included here to provide a direct cell choice comparison to lower density nanodot stripes. We settled on exponential surface coverages given by $M^{i}$ where $M=3$ (and alternatively 3.33) and $i=[0, 1, 2, 3, 4, 5, \ldots, n]$. For each of the three nanodot square sizes; (i) 200 × 200 nm^2^, (ii) 400 × 400 nm^2^ and (iii) 800 × 800 nm^2^, we then calculated the horizontal (x) and vertical (y) spacing between the nanosquares. The nanodot spacing for the arrays dictates the surface coverage density per stripe. To produce linear arrays the spacing dimensions is such that x = y = center–to–center spacing (or pitch). The spacing was calculated using the equations:

$$x=y=k+s [1]$$

$$D=\frac{k^{2}}{\left( k+s \right)^{2}} [2]$$

$$s=\frac{-2kD\pm\sqrt{2kD^{2}-4D(Dk^{2}-k^{2})}}{2D} [3]$$

where, k is the nanodot width, s is the nanodot–to–nanodot gap size and D is the desired surface coverage density. Ultimately, the final densities were slightly adjusted for even distribution and the maximum nanodots coverage that can be achieved with our nanodot fabrication process, which is ~44%.

Squares of the desired nanodots were drawn as instances on L-Edit and arrayed to match different densities producing 40 µm × 400 µm stripes. Each design consists of two stripes with five alternating repeats covering a total area of 400 × 400 µm^2^. Lower and higher coverage stripes were then paired and repeated accordingly as shown in Table 1 below. The number of possible different pairs from these coverages is a combination calculation, _a_C_b_ where a = 7 and b = 2 producing 21 different pairs, across three different nanodot sizes, totaling 63 unique combinations in the NSAs. To evaluate directional persistence, we added three step gradient arrays made by stair-casing the 5 different density nanodot stripes in increasing order.

**Table S1:** The 21 different stripe density combinations used in the NSA showing the concentration differences, fold change and dynamic ranges in each pair.

|  | **Density A [%]** | **Density B [%]** | **Average density [%]** | **Density Difference [%]** | **Fold change [B/A]** | **Dynamic range [O.M.]** |
| --- | --- | --- | --- | --- | --- | --- |
| 1 | **0** | **1** | 0.5 | 1 | **∞** | - |
| 2 | **0** | **3** | 1.5 | 3 | **∞** | - |
| 3 | **0** | **10** | 5 | 10 | **∞** | - |
| 4 | **0** | **30** | 15 | 30 | **∞** | - |
| 5 | **0** | **44** | 22 | 44 | **∞** | - |
| 6 | **0** | **100** | 50 | 100 | **∞** | - |
| 7 | **1** | **3** | 2 | 2 | **3** | 0.477 |
| 8 | **1** | **10** | 5.5 | 9 | **10** | 1.000 |
| 9 | **1** | **30** | 15.5 | 29 | **30** | 1.477 |
| 10 | **1** | **44** | 22.5 | 43 | **44** | 1.643 |
| 11 | **1** | **100** | 50.5 | 99 | **100** | 2.000 |
| 12 | **3** | **10** | 6.5 | 7 | **3.3** | 0.523 |
| 13 | **3** | **30** | 16.5 | 27 | **10** | 1.000 |
| 14 | **3** | **44** | 23.5 | 41 | **14.7** | 1.166 |
| 15 | **3** | **100** | 51.5 | 97 | **33.3** | 1.523 |
| 16 | **10** | **30** | 20 | 20 | **3** | 0.477 |
| 17 | **10** | **44** | 27 | 34 | **4.4** | 0.643 |
| 18 | **10** | **100** | 55 | 90 | **10** | 1.000 |
| 19 | **30** | **44** | 37 | 14 | **1.5** | 0.166 |
| 20 | **30** | **100** | 65 | 70 | **3.3** | 0.523 |
| 21 | **44** | **100** | 72 | 56 | **2.3** | 0.357 |

**Figure S1**: The 63 different combinations for the NSA designs plus three step gradient arrays, shown as they were arranged on the design mask. Asterisks denotes designs that were repeated to fit the seven by ten mask set-up.

## NSA Si Wafer fabrication process flow

**Figure S2**: Fabrication process flow for making the silicon (Si) wafer serving as the master mold for patterning the nanodot stripe assays (NSAs) by lift-off nanocontact printing. The schematic shows a nanodot array adjacent to a stripe with 100 % coverage formed by a deep trench and a stripe with 0 % coverage formed by an area without any pattern at the center. **A–D:** Nanodots were patterned using electron beam lithography (EBL) in ZEP 520a resist, followed by development and reactive ion etching (RIE) with a SF_6_/C_4_F_8_ plasma to a depth of 200 nm into the silicon, and resist stripping **E–H:** Stripes with 100% coverage formed by deep trenches were patterned using a positive Shipley S1813 resist using UV direct writing. Following development of the resist, channels were etched using deep reactive ion etching (DRIE), followed by resist stripping.

## NSA designs overview on Si mold

Here we present an overview image of the different NSA designs on the Si mold. Inserts show enlarged images for selected nanodot stripe designs.


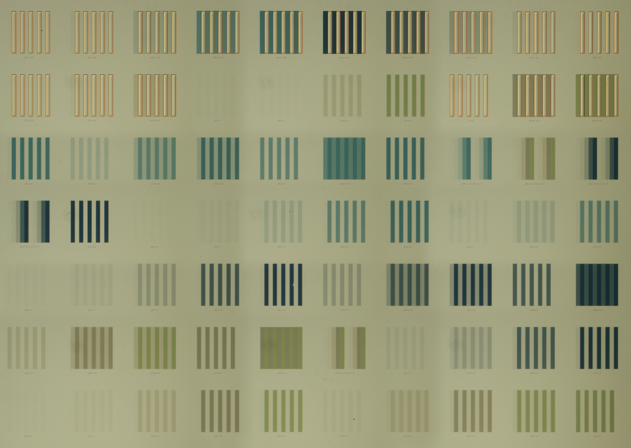


**A**


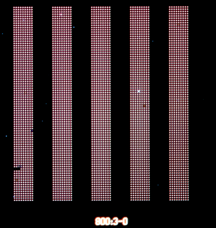

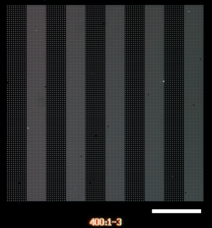

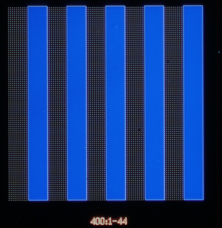


**D**

**C**

**B**

**B**

**C**

**D**

**Figure S3**: The NSA designs on the Si master mold. An overview of all the designs is shown in **A)**. Inserts **B)** through **D)** show zoomed in nanodot stripe pairs highlighting the nanodot features and different stripe densities. Notice that densely packed nanodot arrays appear as ‘homogeneous stripes’ at the low magnification of the images shown in **A** (10X). the observed colors are due light diffraction and reflection phenomena as the Si wafer was not colored. Scale bar: **A)** is 400 µm and **B)** is 100 µm.

## Mold replication and lift-off nanocontact printing

**
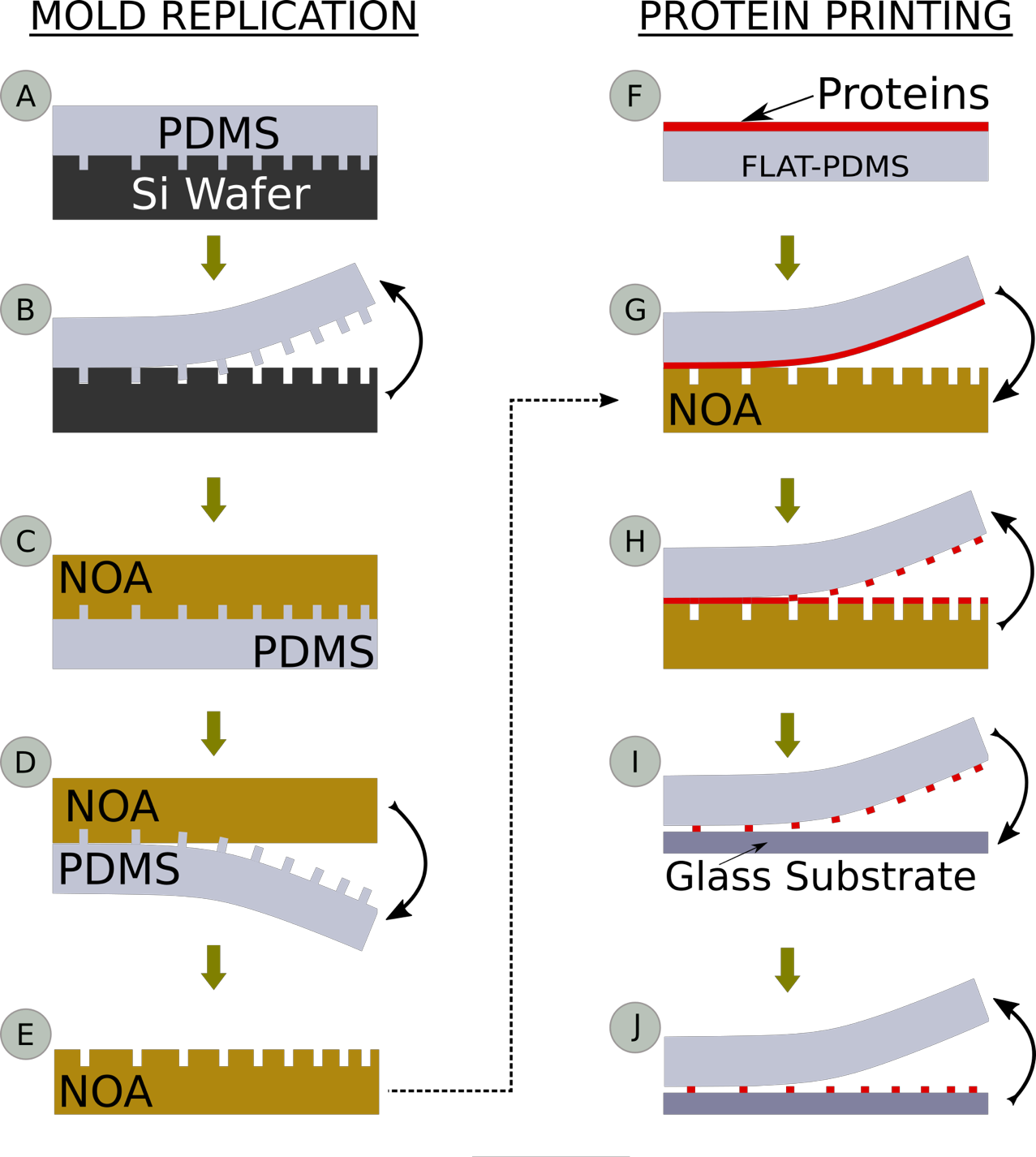
**

**Figure S4**: Double mold replication and lift-off nanocontact printing procedure showing a nanodot array next to a deep trench. Stamps were replicated from the original Si mold to a flexible intermediary PDMS mold and then into rigid NOA-63 stamps **[A–E]**. Proteins were then inked in **[F]**, lifted-off in **[G–H]** with the plasm-activated NOA stamp and finally printed onto the plasma-treated glass cover slip **[I–J]**. This schematic shows a deep etched gap corresponding to a stripe showing an NSA pair with 100% coverage versus a nanodot array. Otherwise, in combinations made of two alternating nanodot arrays, the gap would be represented by nanodots at a different density.

## NSA data extraction procedure


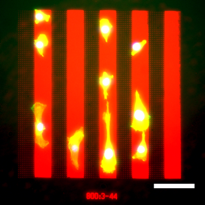


**A**


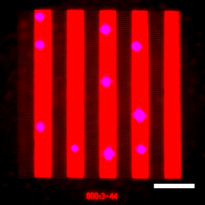


**B**


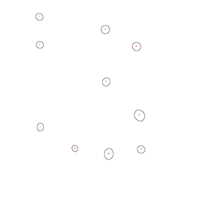


**C**

X-axis

Y-axis

**Figure S5**: Data extraction procedure. **A)** This is a composite image for the netrin-1 proteins (red), cells (green) and nucleus (blue) on design 800:3–44 after performing the 18 h cell migration assay. This NSA illustrates a 100% cell preference since all ten cells accumulated on the 44% stripe at the end of the assay. **B)** The cell nucleus was considered as the cell position and overlayed on prints. **C)** Image J was used to extract the coordinates (horizontal, vertical) of the nuclei in the identified region of interest. Scale bar is 100 µm.

## Cell survival and proliferation on RS-coated substrates

**
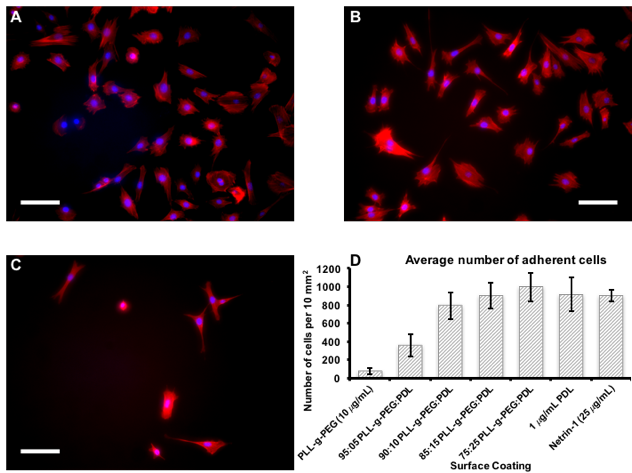
**

**Figure S6**: Cell densities on RSs of different cell-surface affinities quantifies the substrate’s degree of growth permissiveness. **A) – C)** are representative images of adherent cells on glass surfaces with different coatings: **A)** 1 µg/mL PDL, **B)** 90:10 %PEG:%PDL (from stock solutions at 10 µg/mL), and **C)** 100:0 %PEG:%PDL. **D)** The number of adherent cells on the glass substrates coated with different RS mixtures varied as a function of the PDL concentration. Different ratios of %PEG:%PDL yield different cell-surface affinities hence varying the permissiveness of the surface. The lowest cell-surface affinity condition (PEG, 10 µg/mL) has the least cells. Expectedly, the density of cells increases with an increase in PDL concentrations matching the direction of increasing cell-surface affinity. Error bars indicate standard deviation (N ≥ 3). Scale bars are 100 µm.

## Complementary cell migration data

The following plots reveal cell navigation choices on different RSs as well as on nanodots with different dimensions. The fraction of cells (percentage) on the higher density stripe on the NSA designs is presented for each design pair. No cells were found on designs without a column bar.

**Figure S7**: Haptotaxis choices on netrin-1 and control NSAs backfilled with low and high cell-surface affinity RS. These plots show cell preferences averaged across the three different nanodot sizes. **A)** A RS with a low cell-surface affinity (*i.e.* 100% PEG) results in identical response between netrin-1 and the control (IgG). In this condition, the RS is dominated by PEG which has a low cell-surface affinity; therefore, cells were repelled (by the low cell-surface affinity RS) onto the printed netrin-1 cue and even onto the non-cell binding IgG proteins in the control experiments. **B)** A high cell-surface affinity (75:25 %PEG:%PDL) RS impedes migration entirely by masking the presence of the attractive netrin-1 cue. Therefore, in both A and B, the observed response is modulated by the RS, not the netrin-1 cue, which can be misleading in the absence of proper experimental controls. Error bars indicate the standard error of the mean.

**Figure S8**: NSA migration choices of C2C12 myoblasts on netrin-1 nanodot stripes backfilled with 90:10 %PEG:%PDL as the RS. Generally, cells preferred the netrin-1 stripes with higher netrin-1 concentrations while in the control groups, the cells show no clear preference, i.e. cells were randomly distributed. This response applies to all designs regardless of the nanodot sizes. Error bars indicate the standard error of the mean.

**Table S2**: Migration preference scores, standard error of the mean (SEM) and p-values for C2C12 myoblasts on netrin-1 and control NSA backfilled with 90:10 %PEG:%PDL RS, across nanodot sizes. P-values were obtained from a two-tailed t-test and compare migration choices on netrin-1 versus on control (IgG) NSA. Values p< 0.05 are in bold font

| **NSA Pairs** | **Netrin-1** | | **Control (IgG)** | | **p-values** |
| --- | --- | --- | --- | --- | --- |
|  | **Preference score (%)** | **S.E.M (%)** | **Preference score (%)** | **S.E.M (%)** |  |
| 30 vs. 44 | 54.0 | 4.5 | 54.4 | 2.9 | 0.77 |
| 44 vs. 100 | 55.3 | 4.7 | 55.6 | 10 | 0.63 |
| 1 vs. 3 | 64.3 | 8.1 | 46.1 | 6.0 | **0.03** |
| 10 vs. 30 | 70.2 | 3.8 | 55.8 | 5.3 | **0.01** |
| 3 vs. 10 | 78.9 | 3.8 | 43.2 | 5.7 | **8 × 10^-7^** |
| 30 vs. 100 | 67.3 | 7.4 | 35.5 | 7.7 | **2 × 10^-2^** |
| 10 vs. 44 | 82.2 | 2.9 | 45.1 | 8.6 | **2 × 10^-6^** |
| 1 vs. 10 | 90.3 | 7.8 | 53.2 | 4.7 | **9 × 10^-3^** |
| 3 vs. 30 | 84.8 | 2.8 | 60.0 | 6.0 | **7 × 10^-6^** |
| 10 vs. 100 | 80.8 | 5.5 | 52.9 | 23 | **3 × 10^-3^** |
| 3 vs. 44 | 79.7 | 3.5 | 50.6 | 5.2 | **9 × 10^-6^** |
| 1 vs. 30 | 86.1 | 2.1 | 63.8 | 6.9 | **5 × 10^-4^** |
| 3 vs. 100 | 83.2 | 3.2 | 43.5 | 14 | **5 × 10^-5^** |
| 1 vs. 44 | 82.0 | 2.2 | 58.5 | 11 | **4 × 10^-6^** |
| 1 vs. 100 | 86.5 | 2.8 | 36.8 | 11 | **7 × 10^-7^** |
| 0 vs. 1 | 65.2 | 17 | 47.6 | 4.9 | 0.98 |
| 0 vs. 3 | 63.6 | 6.0 | 47.2 | 6.1 | **4 × 10^-3^** |
| 0 vs. 10 | 93.5 | 2.5 | 56.3 | 5.4 | **3 × 10^-7^** |
| 0 vs. 30 | 85.4 | 3.8 | 53.3 | 9.4 | **1 × 10^-4^** |
| 0 vs. 44 | 84.8 | 1.9 | 33.9 | 7.1 | **4 × 10^-12^** |
| 0 vs. 100 | 80.5 | 1.7 | 36.4 | 20 | **8 × 10^-15^** |

## Cell migration data for scored cells

**Table S3**: The total number of cells observed and scored under the three different RS conditions.

|  | **Reference Surface [%PEG: %PDL]** | | | | | | |
| --- | --- | --- | --- | --- | --- | --- | --- |
|  | **100:0** | | **90:10** | | **75:25** | | |
| **NSA Pairs** | **Netrin-1** | **Control** | **Netrin-1** | **Control** | **Netrin-1** | **Control** |  |
| **00 vs. 01** | 10 | 0 | 23 | 147 | 241 | 34 |  |
| **01 vs. 03** | 3 | 0 | 42 | 102 | 162 | 44 |  |
| **00 vs. 03** | 0 | 2 | 55 | 127 | 194 | 34 |  |
| **03 vs. 10** | 12 | 0 | 128 | 95 | 195 | 129 |  |
| **01 vs. 10** | 4 | 6 | 113 | 109 | 217 | 30 |  |
| **00 vs. 10** | 12 | 4 | 124 | 142 | 209 | 76 |  |
| **30 vs. 44** | 131 | 84 | 411 | 125 | 147 | 127 |  |
| **10 vs. 30** | 30 | 38 | 252 | 104 | 173 | 75 |  |
| **03 vs. 30** | 78 | 81 | 264 | 100 | 271 | 123 |  |
| **01 vs. 30** | 42 | 51 | 388 | 58 | 307 | 88 |  |
| **00 vs. 30** | 37 | 57 | 302 | 60 | 244 | 70 |  |
| **10 vs. 44** | 113 | 49 | 376 | 51 | 189 | 73 |  |
| **03 vs. 44** | 159 | 63 | 315 | 89 | 209 | 100 |  |
| **01 vs. 44** | 97 | 142 | 528 | 53 | 194 | 94 |  |
| **00 vs. 44** | 77 | 88 | 501 | 56 | 195 | 101 |  |
| **44 vs. 100** | 139 | 115 | 262 | 45 | 166 | 68 |  |
| **30 vs. 100** | 96 | 145 | 173 | 31 | 189 | 67 |  |
| **10 vs. 100** | 71 | 61 | 359 | 17 | 144 | 53 |  |
| **03 vs. 100** | 80 | 70 | 285 | 23 | 189 | 38 |  |
| **01 vs. 100** | 41 | 94 | 325 | 19 | 157 | 62 |  |
| **00 vs. 100** | 99 | 153 | 154 | 27 | 150 | 28 |  |
